# Heavy-Duty Engine Emissions: Response

**Published:** 2004-09

**Authors:** Janet Arey

**Affiliations:** Department of Environmental Sciences, Air Pollution Research Center, University of California, Riverside, Riverside, California, E-mail: janet.arey@ucr.edu

In his letter, Schaeffer concludes that because of the ongoing changes in diesel technology, “establishing standardized reference materials [of diesel exhaust particles (DEPs)] will be particularly challenging.” As amply illustrated by the work of DeMarini et al. (2004) and Singh et al. (2004), which prompted my commentary (Arey 2004), the effort is worth making because multidisciplinary studies on representative DEP samples are needed if meaningful assessments of the health hazards associated with DEPs are to be made. DeMarini et al. (2004) and Singh et al. (2004) highlighted the chemical, physical, and biological differences between two widely used DEP samples, one mainly studied for pulmonary toxicity and the other for genotoxicity; before their studies, the chemical composition and biologic activity of the samples had not been compared.

In his letter, Schaeffer describes the Advanced Collaborative Emissions Study (ACES), an important diesel assessment project currently in the planning stage by the Health Effects Institute (Boston, MA). Perusing Warren’s presentation on the project (Warren 2004) cited by Schaeffer, I found that the utility of standard reference materials that allow for collaborations and exhaustive characterization of DEPs is reinforced by several issues Warren highlighted; for example, which of the “794 measurements under consideration” should be made; what should the results be compared to; and what health effect testing should be conducted? Until we fully understand the mechanisms of action of diesel and ambient particles that are involved in their adverse health effects, we need more multidisciplinary, collaborative efforts to study samples that can be shared among researchers.
